# Automatic Roll-Profile Positioning Detection System Based on Contact Sensor

**DOI:** 10.3390/s24237606

**Published:** 2024-11-28

**Authors:** Jiali Zheng, Huagui Huang, Qiwei Hu

**Affiliations:** 1 College of Mechanical Engineering, Yanshan University, Qinhuangdao 066004, China; zjl1996@stumail.ysu.edu.cn (J.Z.); hu13892606189@163.com (Q.H.); 2 National Engineering Research Center for Equipment and Technology of Cold Strip Rolling, Yanshan University, Qinhuangdao 066004, China

**Keywords:** roll profile, contact sensor, error analysis, detection, machine vision

## Abstract

Based on the traditional saddle instrument, a portable roll-profile measuring device based on a contact sensor is designed and optimized. The positioning module is added via the machine vision method, which enables the automatic reading of measurement points. The measurement accuracy of the device is 1 μm. Possible errors caused by the process of installing the roll-profile meter are analyzed, and the installation requirements of the measuring device are provided. On this basis, the compensation method for roll measurement with the roll profile is also improved. The accuracy of the measuring device is verified by measuring the roll on the spot, and the measurement error of the flat roll is 0.02 mm. When measuring the roll crown, the measurement accuracy is improved by 69% after the compensation algorithm is applied. The measurement results meet industrial measurement requirements, which is highly significant to the intelligent operation and maintenance of the roll.

## 1. Introduction

As the roller is an irreplaceable part of the rolling production process, its excellent surface quality and roll profile are important for producing high-quality strip steel. Currently, the working environments of rollers can be relatively poor, especially for those used on hot-rolling production lines [1], and replacement and damage are relatively frequent. The cost of roller maintenance account for 10–15% of the total production cost [2,3], and the wear on the working roller is more significant than that on the supporting roller [4]. After rolling about 2000 tons of strip steel on a hot-rolling production line in a steel mill, off-machine roll grinding is carried out. Under some rolling schedules, the roll wear and profile can meet the production requirements of other types of strip steel. In order to reasonably plan the use of a roll [5], it is necessary to detect the roll profile and wear degree of the roll.

At present, widely used roll detection methods include machine vision [6], eddy current sensors [7], laser ranging sensors [8], optical fiber sensors [9], and roll profiles. Machine vision is mainly used to detect surface defects on rollers. This method is also suitable for analyzing the surfaces of cylinders [10]. A camera and light source are used to collect the surface features, and when those data are combined with OTSU threshold segmentation, canny operator edge detection, morphological processing, and other image-processing algorithms, defects can be identified [11]. Xu et al. [12] also restored the three-dimensional morphology of roller surface defects via image fusion, which improved the quality of defect detection images. Long et al. [13] also proposed a high-precision roller detection method based on polarization sensitivity. This method can effectively reduce the drift of the metal roller surface and the influence of stray light and obtain a better roller surface image. Eddy current sensors are mainly used to detect roll damage and other performance aspects. Chen et al. [14] studied a non-contact method based on a pulsed eddy current to evaluate the performance degradation of remanufacturing work rolls. The method can quantitatively evaluate the mechanical properties of remanufacturing work rolls. Rashidi et al. [15] proposed an on-line wear measurement method based on a linear distance sensor for HPGR rolls and designed a measuring device. Methods of calculating wear and possible error sources were discussed. The feasibility of the method was verified experimentally, but it can only be used to detect wear on small-diameter rolls. Guo et al. [16,17,18,19,20] carried out non-contact monitoring of roll wear based on optical fiber sensors, compensated for axis deviation during the measurement process, corrected the error based on a BP neural network, and used an interpolation method and numerical filtering for signal processing. The influence of random noise and roller bearing vibration was effectively reduced, and the detection accuracy and resolution were greatly improved. However, the designed device occupies a large area and is inconvenient to move, and so it is used for on-line detection. Teir et al. [21] used a piezoresistive silicon microprobe sensor in a device for the detection of roll surface roughness. The sensor has a fast measurement speed and can realize the on-line detection of roll roughness, but the measurement range is small. The roll profile is a widely used detection tool in steel mills. The advantage of the roll profile is that it is portable and uses a contact measurement probe. It is less affected by external interference factors. The measurement process maintains resonance with the roll and can reduce certain errors. British Steel [22] proposed a portable roll-measuring instrument that can be used independently without relying on a roll-grinding workshop. The whole device is a semi-arc, and three contact probes are distributed to measure changes in roll diameter. Magdeev [23] manufactured a series of portable instruments with an independent power supply. The instruments were used to detect the actual sizes of large-diameter rolls (those with a diameter greater than 500 mm), and a roll wear correction method was introduced. The current roll-profile detection method and measuring device cannot meet the requirements of portability, versatility, and simple operation. A roll-profile measuring device based on a linear sensor can only measure rolls with small diameters. A measuring device based on an optical fiber sensor needs to move on a longer guide rail, which requires greater measurement space. The process of measuring the roll profile is complicated, and the measurement points need to be manually positioned.

Based on the design appearance of the roll profile, this study simplifies the detection of the roll profile and improves the measurement efficiency. The measuring device is based on machine vision and a laser ranging sensor. It consists of a positioning module and a measuring module with a feature ruler. It can automatically read and store measurement point data and draw the roll curve. Its measurement accuracy is high, which is of great significance for improving the quality and yield of the strip.

## 2. Measuring Device Design

### 2.1. Measurement Requirements

Because the roll is in circular motion when it works, the heat and force from the outside world are the same at each point on each circumferential section during a cycle of the machine. Therefore, this study is based on the premise that the roll profile and wear of each point on the same circumferential surface of the roll are the same.

Due to the different roll sizes on the production line, the roll diameter difference of different passes in the same production line can reach 200 mm. Therefore, the roll profile detection device must be adjustable and able to adapt to different sizes of rolls. Moreover, the measuring device should be as convenient as possible to carry and relatively small.

**Figure 1:**
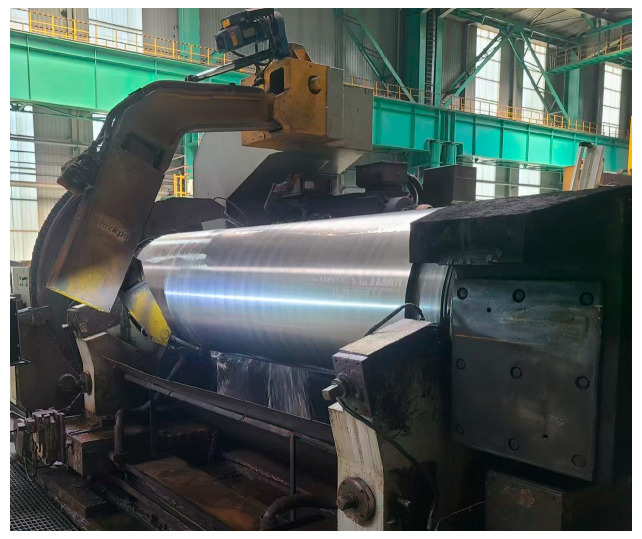
Roll-profile measuring arm of grinding roll workshop.

Investigations have shown that roll-profile measurement does not require measuring points on the entire roll, i.e., it is only necessary to measure a point at a certain distance. At present, the detection equipment used in the rolling mill comprises a saddle instrument and the measuring arm on the grinding machine in the grinding workshop. The automation of the saddle instrument is poor. It is necessary to manually mark measurement points on the roll before detection and then move the saddle instrument to these points for numerical measurement. The measuring arm on the grinding machine has high measurement accuracy, but preparing it before detection is cumbersome. It is necessary to hoist the roll onto the grinding machine for clamping and then perform centering and measurement, as shown in Figure 1.

### 2.2. Principle of Measurement

**Figure 2:**
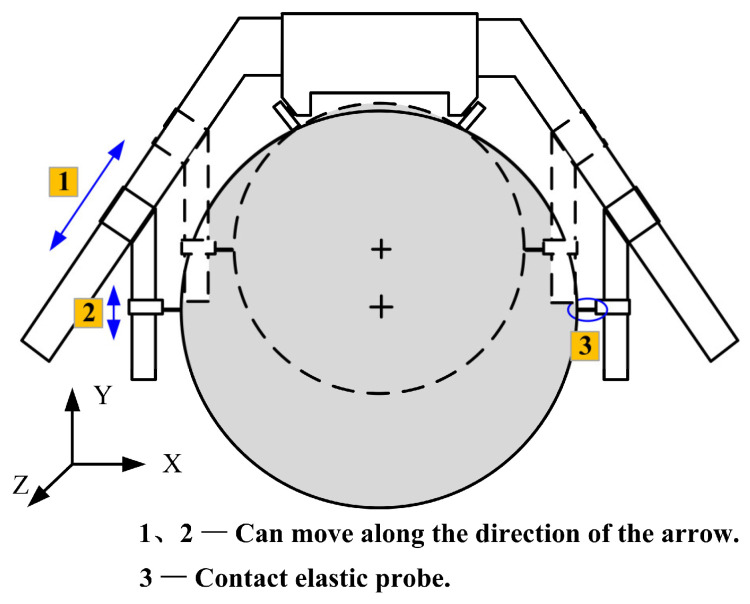
Measurement principle of roll-profile measuring device.

**Figure 3:**
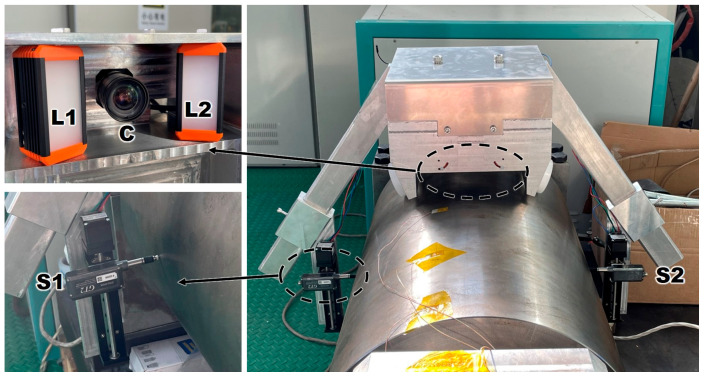
Roll-profile measuring device composition.

The measuring principle of the device is shown in Figure 2. The roll-diameter measuring range of the measuring device is 300–500 mm. The whole measuring device can be divided into a positioning module and a measuring module. The measuring device opens to three degrees of freedom in three directions, and the device can freely adjust the measuring position to adapt to different roll sizes. The whole measuring device is shown in Figure 3. The movement of the measuring device along the Z direction of the roll body is realized using four nylon bearing wheels. The nylon material does not easily deform or slip when sliding on the roll surface. Movement along the circumferential direction of the roll can be realized by adjusting the position of the sliding positioning plate on the measuring arm, and movement in both the X and Y directions can be realized. Position adjustments in the Y direction can also be realized using the stepper-motor slide guide rail. The slide guide rail has high precision and can accurately adjust the measurement position. By adjusting the position of the sliding positioning plate and the slide guide rail, the position changes in directions 1 and 2 in Figure 2 can be realized. The measurement module is based on a GT2-H12K sensor; the measuring range is 12 mm. The scalable range of the sensor contact head can also be used to achieve position fine-tuning in the X direction, and the probe fits the roll surface. When the roll profile changes, the sensor probe will expand, and its value will also change, enabling measurement of the roll profile. The measurement accuracy of the sensor is 1 μm, meeting the detection requirements for almost all rolls.

**Figure 4:**
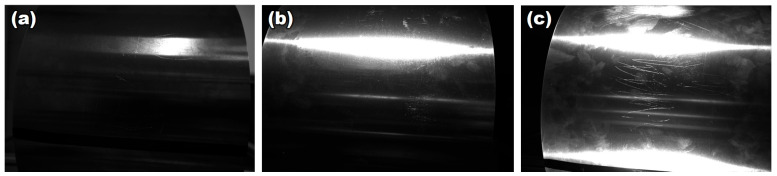
Lighting effect test. (**a**) Natural light; (**b**) single-strip light source; (**c**) double-strip light source.

In order to simplify the positioning method, an automatic positioning module is added inside the measuring device, and measurement point positioning is realized by combining machine vision and a viscous feature scale. Image feature image acquisition is achieved using an area array camera and light source. The equipment was selected as MER-132-43U3C-L camera and HN-0914-2M-C23X lens produced by Daheng Image (Beijing, China), and MV-LLDS-642-28-W strip light source produced by Hikrobo (Hangzhou, China). A characteristic circle is set for each interval distance a on the ruler, where a is the measurement interval and can be set to any value. The lighting results for natural light, a single-strip light source, and a double-strip light source are compared in experiments. The image results collected by the camera are shown in Figure 4. It was found that the use of a double-strip light source can eliminate more reflective effects and result in a clear image of the field of view. Therefore, the double-strip light source was used in further experiments. The feature scale is horizontally pasted onto the upper side surface of the roll to ensure that the scale appears clearly in the field of view of the camera. While moving the measuring device, the ruler will move in the field of view of the camera. When the characteristic circle of the ruler is located in the center of the horizontal direction of the camera’s field of view, it means that the sensor is located at the measurement point and the current sensor data need to be recorded.

**Figure 5:**
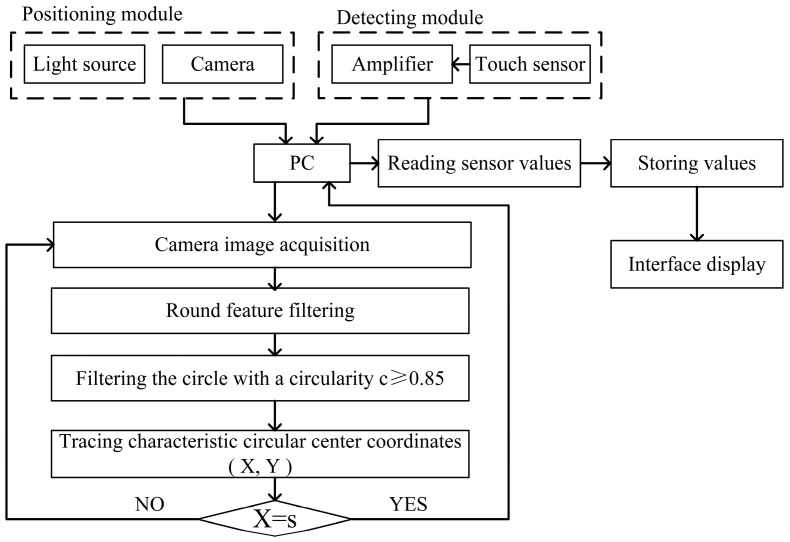
Detection flow chart of roll-profile measurement system.

The process used by the roll-profile measuring device is shown in Figure 5. Both the camera and sensor are connected to a PC, and the PC can immediately obtain images from the camera and the sensor’s reading. As the device is moved, the camera continuously filters the features of the entire field of view and filters out set feature patterns through morphological processing. Roundness information is used to lock the specific feature pattern. When the roundness is greater than 0.85, the feature capture is considered to meet the requirements. The center X coordinates of the feature group are tracked in real time. When the feature pattern reaches the set position in the camera’s field of view, X = s, the PC receives and stores the readings of the current sensor. This process is continuously repeated until detection is completed. After many experiments involving the moving speed of the roll-measuring device, it is determined that the time taken for the measurement point to pass through the camera’s field of view should be more than 1.5 s, that is, the moving speed should be less than 0.14 m/s. Otherwise, the measurement point could be missed because the speed is too fast to capture the feature point. The relative displacement between each measurement point and the initial measurement point is used to obtain the roll profile data.

### 2.3. Device Measurement Interface Design

**Figure 6:**
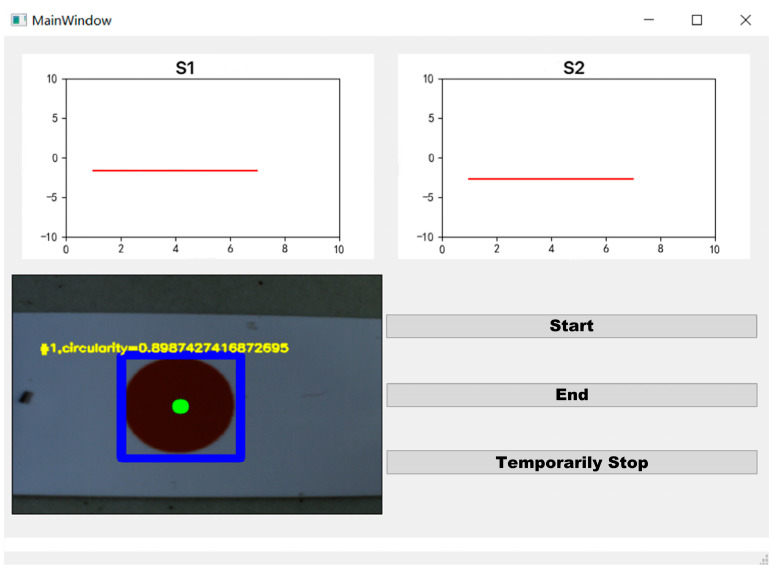
Interface design of detection system.

The interface of the measurement system is shown in Figure 6, and the data curves of sensors S1 and S2 are shown in the above two charts. The dialog box in the lower left shows the camera image, and the right buttons are used to start the measurement, end the measurement, and temporarily stop.

## 3. Detection System Error Analysis

The position of the measuring device may deviate while positioning the measuring point before the measurement and during the measurement process, thus affecting the measurement results. This section lists the various situations that may occur when the sensor position of the measuring device is adjusted; additionally, the measurement error is analyzed and estimated.

**Figure 7:**
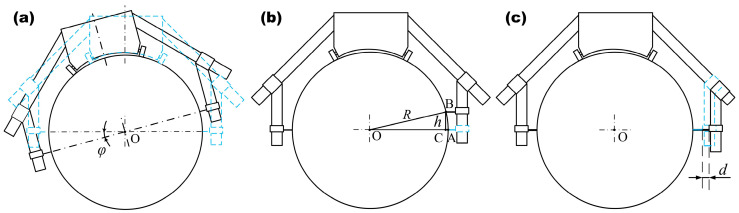
Measurement device installation error analysis. (**a**) Device rotation. (**b**) The measurement position of the unilateral sensor is high. (**c**) Remote position of unilateral sensor.

(1) The roll-profile detection device rotates along the circumferential direction of the roll in the process of moving along the roll body, as shown in Figure 7a. In this figure, the blue dotted line represents the theoretical measurement position of the measuring device, and the black solid line represents the position of the measuring device after offset. The measuring device rotates the angle as a whole, and the central surface of the measuring device continues around the roll’s axis when it rotates. That is, the center of the measured cross-section of the roll is still located on the line connecting the two sensors, and the line connecting the two sensor measurement points is still the diameter of the roll. Therefore, in this case, there is no effect on the measurement results, and the measurement results are still the actual roll profile.

(2) The measuring points of the left and right sensors of the roll profile measuring device are not coplanar with the roll axis, such as when the measuring points of the unilateral and bilateral sensors are higher or lower. Taking a high position of the unilateral sensor as an example, the measurement diagram is shown in Figure 7b. The blue dotted line represents the theoretical measurement position of the sensor, and the measurement point of the right sensor is shifted from point A to point B. The offset distance is h, and the measured error value, that is, the length n of the CA, is $n=R-\sqrt{R^{2}-h^{2}}$. If the offset distance is 1 mm and the roll radius is 300 mm, the measurement error is 16 μm.

(3) The sensors on the left and right sides are not symmetrical in the X direction, as shown in Figure 7c. The blue dotted line represents the theoretical position of the sensor, and the position of the right sensor is farther to the right. Since the probe of the contact sensor is scalable, it is attached to the surface of the roll during the measurement process, and the probe adapts to the roll profile to change the expansion and contraction so that the value changes. The roll profile of each point detected by the measuring device is the change compared to the initial measuring point. Therefore, as long as the sensor value is zeroed at the initial measurement point, the obtained measurement result is still an accurate roll profile result. This situation has no effect on the measurement results of the roll profile.

In summary, when the measuring device is installed, the connection of the measuring points of the sensors on both sides should be coplanar with the axis of the roll to ensure accurate measurement.

## 4. Roll-Profile Compensation

**Figure 8:**
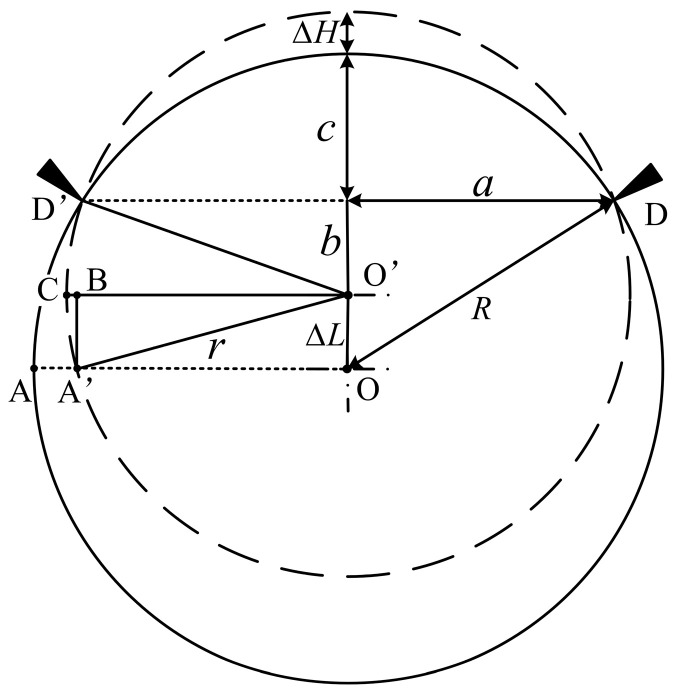
Roll compensation schematic diagram.

Some of the rolls in a production line have concave and convex roll profiles. These kinds of rolls need to be compensated for during measurement; otherwise, they will produce large measurement errors. Therefore, an error compensation algorithm is added to the process of roll measurement with a roll profile. When the roll profile of the roll changes, the horizontal measurement point of the whole device will move in the vertical direction during the measurement process, meaning that the position of the contact ranging sensor cannot be in the same measurement line position, resulting in measurement error. In order to increase the measurement accuracy, a roll-profile change compensation algorithm is introduced. The compensation principle is shown in Figure 8. Points D and D′ are the contact points between roller and detection device. The distance between the rollers of the device is fixed, and the distance between the two points of DD′ is fixed at 2a. The solid-line circle O is the cross-section of the initial measurement point at the edge of the roll. Its radius is R, and the initial radius R is known. The dotted circle O′ is the cross section of a roll after the roll profile changes, and the radius is r. When the roll profile of the roll changes during the detection process, the measurement point changes from point A to point A′, and the theoretical measurement point after changing the roll profile should be point C. Therefore, it is necessary to use the algorithm to compensate the CB segment. Through analyzing the roll-profile curve of the field collection point, it is found that the change in the roll profile occurs when the measurement spacing is $\left|f\left(x+l\right)-f\left(x\right)\right|$. This is a function related to the roll-profile curve, and the allowable roll wear generally does not exceed 0.3 mm. When this value is exceeded, the roll cannot meet the requirements of the machine, and so the input in the program when $\Delta H\geq \left|f\left(x+l\right)-f\left(x\right)\right|$, the measurement system considers that the roll profile changes and compensates according to the change in the value. l is the distance between measuring points.

According to the triangular geometric relationship before and after the roll profile change, the following can be obtained:

(1) $$a^{2}=r^{2}-b^{2}=R^{2}-{\left(\Delta L+b\right)}^{2}$$

The roll radius R of the initial roll profile and the roll radius r after changing the roll profile can be expressed as follows:

(2) r=ΔH+c+b

(3) R=c+b+ΔL

Subtract Equation (2) from Equation (3):

(4) r=R+ΔH−ΔL

Bringing Equation (4) into Equation (1) to obtain the offset of the axis ΔL after the roll profile is changed yields the following:

(5) $$\Delta L=\frac{\Delta H^{2}+2\Delta HR}{2\left(R+\Delta H\pm \sqrt{R^{2}-a^{2}}\right)}$$

The compensation amount BC can be expressed as follows:

(6) $$\mathrm{BC}=r-{\mathrm{OB}}^{\prime }=r-\sqrt{r^{2}-\Delta L^{2}}$$

The compensation amount ΔX is obtained by bringing Equations (4) and (5) into Equation (6):

(7) $$\begin{array}{l} \Delta X=R+\Delta H-\Delta L-\sqrt{{\left(R+\Delta H-\Delta L\right)}^{2}-\Delta L^{2}}\\ =R+\Delta H-\frac{\Delta H^{2}+2\Delta HR}{2\left(R+\Delta H\pm \sqrt{R^{2}-a^{2}}\right)}-\sqrt{\left(R+\Delta H\right)\left(R+\Delta H-\frac{\Delta H^{2}+2\Delta HR}{R+\Delta H\pm \sqrt{R^{2}-a^{2}}}\right)} \end{array}$$

## 5. Detection System Test Experiment

**Figure 9:**
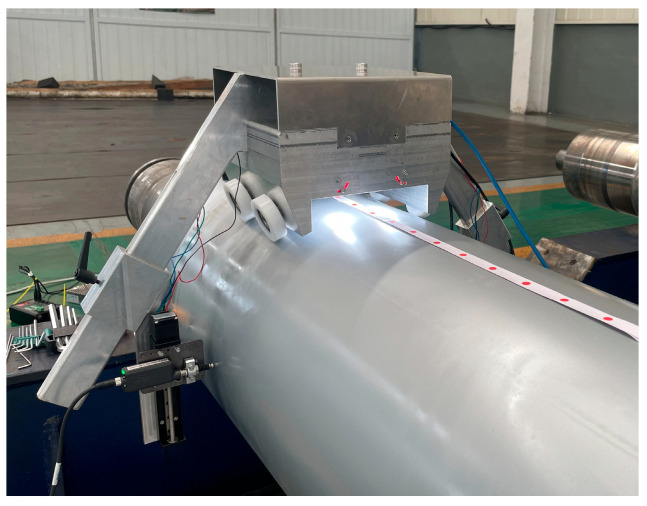
The roll profile detection device performs measurement experiments on the roll.

Experiments were carried out on an electromagnetic heating roll in the laboratory to test the function of the roll-profile measuring device. Figure 9 shows the installation and test position of the roll-profile measuring device on the electromagnetic heating roll. The device is placed above the roll to ensure that the nylon roller contacts the roll surface.

**Figure 10:**
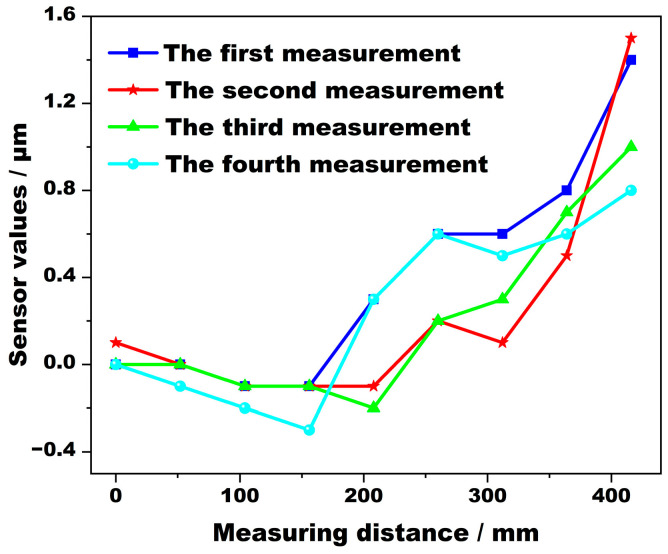
Repeated measurement error curve.

Multiple sets of repeated measurements were performed on the same section of the roll. The results of the four measurements are shown in Figure 10. The measurement results show that the value is accurate to 0.1 μm. The maximum fluctuation of the measurement results at the same point is 0.94 μm, which is less than the measurement accuracy of 1 μm. The repeatability error is small, and the measurement results are stable.

The device was taken to a steel mill for an on-site roll-profile detection test. The rolls measured included flat and crowned rolls. The measuring device can be easily used. The detection accuracy of the on-site roll profile was required to be less than 0.02 mm, and the measurement spacing was 52 mm.

**Figure 11:**
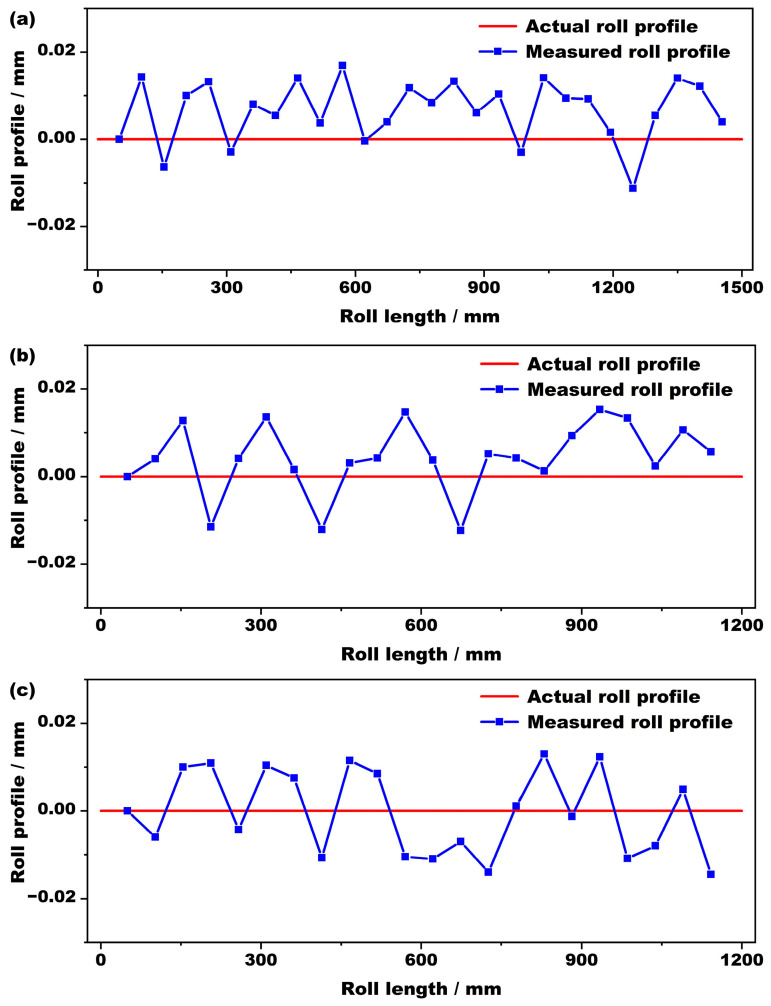
The measurement results of flat rolls with different diameters. (**a**) Diameter 480 mm; (**b**) diameter 400 mm; (**c**) diameter 350 mm.

In order to verify the feasibility of using the roll-profile measuring device to measure rolls of different sizes, flat rolls of different diameters were measured. The roll profile measurement results are shown in Figure 11. Figure 11a shows the roll profile measurement results for rolls with a diameter of 480 mm and a length of 1500 mm, a diameter of 400 mm and a length of 1200 mm, and a diameter of 350 mm and a length of 1200 mm, respectively. The red curve in the figure is the reference curve of the actual roll profile of the roll, and the blue curve is the test result of the measuring device. There are some fluctuations in the roll profile curves of the three types of rolls. This was due to noise and vibration interference at the site and the unstable shaking produced by the measuring device and measured rolls. Placing the roll-profile measuring device on the roll and co-oscillating it with the roll at the same frequency can eliminate the influence of some noise. However, weak noise when measuring some parts is unpredictable, and so there will be some jitter in the measurement curve. It can be seen from the measurement results in the figure that the maximum measurement error is 0.018 mm, which meets the error requirement range for field measurements.

**Figure 12:**
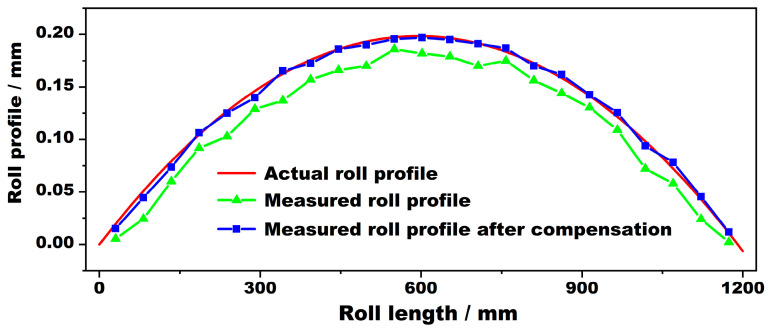
Measured profile curves of positive-crown roll before and after compensation.

In order to test the accuracy of the roll-profile compensation program, a test was carried out on a crowned roll. The measured roll crown was 0.4, the roll body length was 1200 mm, and the roll diameter was 400 mm. The roll-profile measurement results are shown in Figure 12. The measured roll profile without error compensation is shown by the green curve in the figure. The overall measured value is small, and the maximum measurement error can reach 0.052 mm. The measured roll profile after using the error compensation algorithm is shown by the blue curve in the figure; the setting in the compensation program was 0.037. It can be seen from the diagram that the accuracy of the compensated roll profile is obviously improved, as it more closely resembles the actual roll profile curve in the diagram (the reference, shown in red). The measurement accuracy after compensation can be controlled to within 0.016 mm, and the accuracy of the compensated roll-profile measurement result is 69% higher than that of the uncompensated roll-profile measurement result. This method can accurately measure the profile curve of the roll, meeting industrial requirements.

## 6. Conclusions

In this study, a portable roll-profile measuring instrument based on a contact sensor was designed. The measurement accuracy of the device is as fine as 1 μm. The positioning module is added using the machine vision method. The best scheme for the light source was verified experimentally, and automatic reading and storage of measurement point data were realized. Error analysis was carried out for several problems that may occur during the installation of the roll-profile measuring instrument. Finally, it was determined that the connection of the measuring points on both sides of the sensor and the axis of the roll should be coplanar to meet the installation requirements. For detection of a roll profile, a compensation method for the roll profile was also introduced. The stability of the device was tested in repeated measurement experiments, which showed that the repeatability error was less than 1 μm. Using the measurement error of the on-site roll detection device, flat and crowned rolls were selected for roll-profile measurement. The measurement error for the flat roll was within 0.016 mm. Additionally, the reliability of the roll compensation method was verified for measuring crowned rolls. The experimental results show that the measurement accuracy is improved by 69% after using the compensation algorithm, and the measurement errors are in line with the error requirements. The system has the advantages of portability, stability, and high measurement accuracy and can be used for on-site roll measurement. These results are of great significance for optimizing the practicability of the rolling process, and they lay a foundation for the intelligent operation and maintenance of rolls.

## Data Availability

The data presented in this study are available on request from the corresponding author due to the data being obtained through experiments and not being publicly available.
